# Lesion Load May Predict Long-Term Cognitive Dysfunction in Multiple Sclerosis Patients

**DOI:** 10.1371/journal.pone.0120754

**Published:** 2015-03-27

**Authors:** Francesco Patti, Manuela De Stefano, Luigi Lavorgna, Silvia Messina, Clara Grazia Chisari, Domenico Ippolito, Roberta Lanzillo, Veria Vacchiano, Sabrina Realmuto, Paola Valentino, Gabriella Coniglio, Maria Buccafusca, Damiano Paolicelli, Alessandro D’Ambrosio, Patrizia Montella, Vincenzo Brescia Morra, Giovanni Savettieri, Bruno Alfano, Antonio Gallo, Isabella Simone, Rosa Viterbo, Mario Zappia, Simona Bonavita, Gioacchino Tedeschi

**Affiliations:** 1 Department G.F. Ingrassia, Section of Neurosciences, University of Catania, Catania, Italy; 2 Department of Medical, Surgical, Neurological, Metabolic and Aging Sciences, Second University of Naples, Naples, Italy; 3 Department of Neurological Sciences, University ‘Federico II,’ Naples, Italy; 4 Department of Experimental Biomedicine and Clinical Neurosciences-University of Palermo, Palermo, Italy; 5 Department of Medical Sciences, Institute of Neurology, University “Magna Graecia”, Catanzaro, Italy; 6 Department of Neurology, “Madonna delle Grazie” Hospital, Matera, Italy; 7 Department of Neurosciences, Psychiatry and Anaesthesiology, University of Messina, Messina, Italy; 8 Department “Scienze Mediche di Base, Neuroscienze e Organi di Senso”, University of Bari, Bari, Italy; 9 Biostructure and Bioimaging Institute, National Research Council, Naples, Italy; 10 Neurological Institute for Diagnosis and Care “Hermitage Capodimonte”, Naples, Italy; Kessler Foundation Research Center, UNITED STATES

## Abstract

**Background:**

Magnetic Resonance Imaging (MRI) techniques provided evidences into the understanding of cognitive impairment (CIm) in Multiple Sclerosis (MS).

**Objectives:**

To investigate the role of white matter (WM) and gray matter (GM) in predicting long-term CIm in a cohort of MS patients.

**Methods:**

303 out of 597 patients participating in a previous multicenter clinical-MRI study were enrolled (49.4% were lost at follow-up). The following MRI parameters, expressed as fraction (f) of intracranial volume, were evaluated: cerebrospinal fluid (CSF-f), WM-f, GM-f and abnormal WM (AWM-f), a measure of lesion load. Nine years later, cognitive status was assessed in 241 patients using the Symbol Digit Modalities Test (SDMT), the Semantically Related Word List Test (SRWL), the Modified Card Sorting Test (MCST), and the Paced Auditory Serial Addition Test (PASAT). In particular, being SRWL a memory test, both immediate recall and delayed recall were evaluated. MCST scoring was calculated based on the number of categories, number of perseverative and non-perseverative errors.

**Results:**

AWM-f was predictive of an impaired performance 9 years ahead in SDMT (OR 1.49, CI 1.12–1.97 p = 0.006), PASAT (OR 1.43, CI 1.14–1.80 p = 0.002), SRWL-immediate recall (OR 1.72 CI 1.35–2.20 p<0.001), SRWL-delayed recall (OR 1.61 CI 1.28–2.03 p<0.001), MCST-category (OR 1.52, CI 1.2–1.9 p<0.001), MCST-perseverative error(OR 1.51 CI 1.2–1.9 p = 0.001), MCST-non perseverative error (OR 1.26 CI 1.02–1.55 p = 0.032).

**Conclusion:**

In our large MS cohort, focal WM damage appeared to be the most relevant predictor of the long-term cognitive outcome.

## Introduction

Cognitive Impairment (CIm) has been recognized as an important feature of Multiple Sclerosis (MS), affecting up to 65% patients. CIm, such as memory impairment, reduced information processing speed, attention deficit, impaired executive function, can occur from the early stage of the disease and tends to worsen over time. The prevailing pattern of CIm in MS is represented by attention, processing speed, memory, executive function and visuo-spatial deficits, while language abilities are typically unaffected [1]. In the last years, novel Magnetic Resonance Imaging (MRI) techniques have provided further evidences into the understanding of CIm in MS, highlighting the involvement of both white matter (WM) and gray matter (GM) damage in the development of disability [2, 3].T1-, T2-lesion load (LL) and brain atrophy measures may predict the onset of CIm after several years [4, 5]. Conversely, other studies showed a clear discrepancy between LL and severity of CIm in MS [6].WM abnormalities were weakly correlated with CIm, suggesting that WM abnormalities alone cannot fully explain the extent of clinical symptoms and CIm in MS [7, 8].In the present study, WM, and GM atrophy and WM LL were obtained through a fully automated, operator-independent, multiparametric segmentation method from a large MS population [9, 10]. By using this approach, we recently showed that baseline (BL) GM atrophy and EDSS were the best long-term (9 years follow-up) predictors of clinical disease progression in relapsing remitting (RR) MS patients[11].Considering these findings, the aim of the present study was to investigate the role of WM and GM damage in predicting long term (9 years follow-up) CIm in a large multicenter cohort of MS patients.

## Materials and Methods

### Ethics statement

The study was previously approved by the Ethics Committee (EC) of the Second University of Naples and then by all local EC of each participating center: EC “Federico II” Naples, EC University Hospital Policlinico Palermo, EC University Hospital Policlinico Catanzaro, EC University Hospital Policlinico Messina, EC University Hospital Policlinico Bari, National Research Council, Naples. A written informed consent was obtained from every patient before study initiation.

### Patients

In line with our recent paper, of the initial cohort of 597 MS patients participating to the previous multicenter clinical/MRI research, 303 subjects were enrolled in this 9-year follow-up (FU) study (49.4% lost at FU). The reasons for drop-out were: missing contact information (50.7%), unavailability (24.1%), refusal (20.1%), death (3.4%), and other major medical illnesses (1.7%). There were no significant differences in BL demographic or clinical data between patients lost at FU and those participating in the initial cross-sectional study, except for a minor percentage of RR patients in the dropped-out population (65% RR patients in the dropped out group, 72.4% in the FU group; p <0.02) [11]. Out of 303 subjects, 241 underwent the Neuropsychological (NPS) battery. Furthermore, not all patients performed the whole NPS battery; a slight partial incompleteness was obtained for some test because of the challenging duration of the NPS examination. At BL and at FU, disability was measured by the EDSS and fatigue was evaluated by the Fatigue Severity Scale (FSS). ΔEDSS and ΔFSS represent the variation of these parameters during FU. Nine years after, 42 out of 241(17.4%) RRMS patients converted to Secondary-Progressive (SP) MS (RR→ SP), while the remaining 199 (82.6%) did not change their disease phenotype (RR→ RR).

### MRI imaging

**Fig 1:**
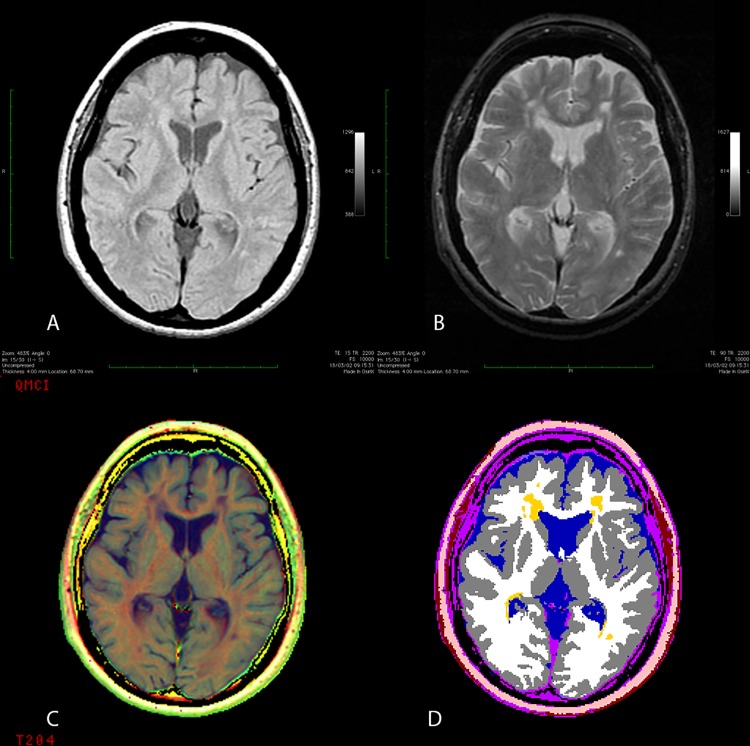
Axial image of a Multiple Sclerosis patient. (A) Proton density-weighted image; (B) T2-weighted image; (C) corresponding multiparametric image; (D) segmented image. In the segmented image, white matter is represented by white, gray matter by gray, cerebrospinal fluid by blue, and lesions by yellow.

At BL, the enrolled patients underwent the MRI protocol described in the previous cross-sectional study [10]. In brief, conventional spin echo sequences were acquired to obtain T1-weighted (TR/TE 600/15 msec, two averages) and dual echo (TR/TE2300/15–90 msec, one average) images, with 90°flip angle and 256 × 192 matrix size. All the studies were segmented using a multispectral, fully automated method, based on relaxometric characterization of brain tissues [9]. The program furnishes complete sets of multifeature images [R1 (= 1/T1), R2 (= 1/T2), proton density (N(H))-based] and segmented images of the following intracranial tissues: cerebrospinal fluid (CSF), WM, GM, abnormal WM. AWM is a WM LL measure as determined by the R1, R2, and N(H) information and morphological characteristics. The relaxometric method used provides GM segmentation not influenced by WM lesions [12]. For each study, a couple of interactive interslice movies of both multifeature and segmented images were produced, and two neuroimaging experts reviewed them (for a maximum of 2 minutes) to detect motion artifacts and segmentation errors due to the imperfect separation of nasal mucosa and vitreous humor from brain tissue. To normalize for head size variability, the volumes were expressed as fraction (f) of intracranial volume. AWM-f is measure of LL. The reduction of WM-f and GM-f indicate respectively WM and GM volume reduction. The increase of CSF-f indicates global brain volume reduction. Fig. 1 shows a transverse slice of a patient with MS and depicts the major steps of the multiparametric method.

### Neuropsychological evaluation (NPS)

At the 9-year FU visit, all patients underwent the same NPS battery, standardized among the participating centers. Tests were administered during daytime, in a quiet room. A brief neuropsychological battery was administered to explore the following cognitive domains.

#### Sustained attention

Symbol Digit Modalities Test (SDMT, number of correct pairings). Patients are presented a series of nine symbols. Every symbol is paired with a single digit, labeled 1–9. In the test page a pseudo-randomized sequence of symbols is presented. Patients have to write the correct digit associated to every symbols as quick as possible in 90 seconds [13]

#### Verbal memory

Semantically related word list test (SRWL):the test consisted of five consecutive immediate free-recall trials (I-R), followed by a 15-min delayed recall trial (D-R) and recognition of 16 words from 4 categories: animals, transportation, vegetables and furniture [14].

#### Executive functions, conceptual reasoning

Modified Card Sorting Test (MCST) was adapted to improve Wisconsin Card Sort Test (WCST), simplifying the task, removing the ambiguity in interpreting responses and providing measure of perseveration. The cards were always presented to each participant in the same order according to the sequence provided by the numbers at the back of the cards. Errors were scored as perseverative if the sorting response was the same category (color, form, number) as the previously incorrect response, or if the sorting response did not change after the patient was told that the rules had changed. An error was scored as not perseverative if the patient followed a sorting response to neither color, form or number (“other” response) with a second “other” response. MCST scoring is based on the number of categories completed and the number of perseverative and non-perseverative errors [15].

#### Information speed processing, working memory

Three-second version of Paced Auditory Serial Addition Test (PASAT) is a test measuring sustained attention and information processing speed. This test is included in the Brief Repeatable Battery of Neuropsychological test (BRB). A series of single digit numbers from a type record are presented to the subject at the rate of one every 3 seconds. The subject is asked to add every digit to the one immediately proceeding. The test is composed by 61 digits, the maximum score is 60 [16].

PASAT and SDMT were corrected using the available normative data for the Italian population. We considered as cut off point a corrected score below the 5th percentile of the normative data [17]. SRWL raw scores were corrected for sex, age and education; cut-off points were calculated using the available normative data [14]. MCST raw scores were corrected for age; a cut off was calculated for every measure using the available internal tolerance limit according to the MCST-Roma version [18].

Higher values indicate better performances in all tests but MCST perseverative and non perseverative error; in these items higher values indicate worse performance. An overall Cognitive Index (CI) grading score was calculated for each patient. Considering the number of standard deviations (SD) below mean of normative values, cognitive tests were graded as follows:0, at or above mean value; 1, below mean but at or above 1 SD below mean; 2, < 1SD below mean but at or above 2SD below mean; 3, <2SD below mean but at or above 3SD below mean; 4, <3SD below mean. These grades were summed to obtain an overall CIm for each patient [19]. CIm was defined as the failure on at least two tests involving at least two different domain (verbal memory, attention/information processing speed and executive functions).

### Statistical Analysis

Age, disease duration, NPS scores and MRI volume data are presented as means and SD (see Table 1). Individual variables were checked for skewness and presence of outliers and the mean MRI parameters of all subjects were adjusted for age, gender and education using a linear regression model. Statistical analysis was performed using STATA 12.0, and a p value < 0.05 was considered statistically significant. Chi square test was used to compare NPS test score and CIm between RR → RR and RR→SP.

**Table 1:** Demographic, clinical characteristics, *adjusted baseline MRI volume (as percentage of whole brain volume) and **neuropsychological test scores of MS patients (mean ± SD).

	RR-RR	RR-SP
**n**	n = 199	n = 42
**Sex**	145 F54 M	29 F13 M
**Age at FU**	43.8 ± 8.9	47.3 ± 9.8
**Age at onset**	27.2 ± 7.9	28 ± 8.8
**Baseline EDSS**	2.1 ± 0.9	3.17 ± 1.0
**DD baseline years**	7.8 ± 6.0	10.2 ± 7.5
**DD FU years**	16.9 ± 6.0	19.3 ± 8.0
**AWM-f**	1.0 ± 1.3	2.1 ± 2.5
**CSF-f**	14.2 ± 4.1	18.0 ± 5.8
**GM-f**	51.9 ± 2.8	49.4 ± 3.6
**SRWL-IR**	41.2 ± 11	34.7 ± 12.4
**SRWL-DR**	9 ± 3.3	9.7 ± 11
**SRWL-REC**	14.2 ± 3.1	14.6 ± 4.8
**SDMT** **	-1.3 ± 1.5	-2 ± 1
**PASAT** **	-1.1 ± 1.2	-2 ± 0.9
**MCST-CAT**	5.4 ± 1.9	4.7 ± 2.2
**MCST-PE**	4.4 ± 7	5.2 ± 6.2
**MCST-NPE**	5 ± 5.6	6.2 ± 6.9

*Adjusted for age, gender and education.

** *z* score.

Several univariable logistic regression were fit considering binomial “impaired yes/no” variable for each NPS as dependent and age, age at onset, CSF-f, AWM-f, GM-f, GWM-f, WM-f, BLEDSS, ΔEDSS, BLFSS, ΔFSS, disease duration, progression to SPMS and gender as independent variables. Variables correlating with outcomes (p < 0.1) in univariable analysis were used as independent variables in logistic stepwise regression, considering p = 0.10 as the critical value for entering or excluding variables in the model. A negative binomial regression analysis was performed to evaluate the correlation with the severity of CIm index score.

## Results

Demographic, clinical, cognitive and MRI data are summarized in Table 1. In Tables 2 and 3 univariable and multivariable analyses are summarized. SDMT scores of 222 MS patients were evaluated. Nineteen patients were excluded from the analysis because their SDMT scores were not available. If compared to the RR→SP, RR→RR group showed higher SDMT scores (p<0.001). The logistic regression step-wise model regarding SDMT showed a significant positive correlation with AWM-f (OR 1.49, CI 1.12–1.97 p = 0.006), EDSS at BL (OR 1.066 CI 1.19–2.32 p = 0.003), ΔEDSS (OR 1.66 CI 1.19–2.32 p = 0.003), ΔFSS (OR1.66 CI 1.4–2.2 p<0.001). This means that for a 1% increase in AWM-f there was a 49% increased odds to be cognitively impaired at this test. Moreover, the EDSS at BL and the variations in EDSS and FSS scores were related to higher impaired scores at SDMT (see Table 3).

**Table 2:** Univariable analysis verbal memory, sustained attention/information processing speed, executive function/conceptual reasoning.

VERBAL MEMORY				SUSTAINED ATTENTION/INFORMATION PROCESSING SPEED		EXECUTIVE FUNCTION/CONCEPTUAL REASONING		
	**SRWL-IR n = 230**	**SRWL-DR n = 230**	**SRWL-REC n = 228**	**SDMT n = 222**	**PASAT n = 217**	**MCST-CAT n = 224**	**MCST-PE n = 199**	**MCST-NPE n = 199**
**Age**	OR 0.99, p = 0.783	OR 1.00, p = 0.896	OR 1.00, p = 0.993	**OR 1.04, p = 0.008**	**OR 1.04, p = 0.009**	OR 1.02, p = 0.318	OR 1.1, p = 0.582	OR 1.04, p = 0.059
	CI 0.9–1.0	CI 1.0–1.1	CI 0.9–1.1	**CI1.01–1.07**	**CI 1.01–1.07**	CI 1.0–1.1	CI 1.0–1.1	CI 1.0–1.1
**Age at onset**	OR 0.98 p = 0.234	OR 0.98, p = 0.401	OR 0.99, p = 0.901	OR 1.01, p = 0.446	OR 1.02, p = 0.164	OR 0.98, p = 0.358	OR1.0 p = 0.82	OR 1.0 p = 0.814
	CI 0.9–1.0	CI 0.9–1.0	CI 0.9–1.1	CI1.0–1.1	CI 1.0–1.1	CI 0.9–1.0	CI 0.9–1.0	CI 0.9–1.0
**AWM-f**	**OR 1.73 p<0.001**	**OR 1.63, p<0.001**	OR 1.27, p = 0.523	**OR 1.59, p<0.001**	**OR 1.39, p = 0.004**	**OR 1.50, p<0.001**	**OR 1.48, p = 0.001**	OR 1.24, p = 0.037
	**CI 1.4–2.2**	**CI 1.3–2.0**	CI 0.6–2.6	**CI1.2–2.1**	**CI 1.1–1.7**	**CI 1.2–1.8**	**CI 1.2–1.9**	CI 1.01–1.52
**CSF-f**	**OR 1.15 p<0.001**	**OR 1.13, p = 0.001**	OR 1.11, p = 0.329	**OR 1.13, p<0.001**	**OR 1.10, p = 0.003**	**OR 1.11, p = 0.001**	OR 1.03, p = 0.382	OR 1.06, p = 0.087
	**CI 1.1–1.2**	**CI 1.1–1.2**	CI 0.9–1.4	**CI1.1–1.2**	**CI 1.03–1.18**	**CI 1.05–1.18**	CI 1.0–1.1	CI 1.0–1.1
**GM-f**	**OR 0.8 p<0.001**	**OR 0.83, p = 0.001**	OR 0.94, p = 0.638	**OR 0.84, p = 0.001**	**OR 0.88, p = 0.007**	**OR 0.87, p = 0.003**	OR 0.94, p = 0.22	OR 0.93, p = 0.177
	**CI 0.7–0.9**	**CI 0.7–0.9**	CI 0.7–1.2	**CI0.8–0.9**	**CI 0.8–0.9**	**CI 0.8–0.96**	CI 0.8–1.0	CI 0.8–1.0
**GWM-f**	**OR 0.9 p = 0.03**	**OR 0.89, p = 0.057**	OR 0.88, p = 0.353	OR 0.95, p = 0.219	OR 0.95, p = 0.263	**OR 0.90, p = 0.032**	OR 1.00, p = 0.921	OR 0.95, p = 0.365
	**CI 0.86–1.03**	**CI 0.8–1.0**	CI 0.7–1.1	CI 0.9–1.0	CI 0.9–1.0	**CI 0.83–0.99**	CI 0.9–1.1	CI 0.9–1.1
**WM-f**	**OR 0.8 p<0.001**	**OR 0.85, p<0.001**	OR 0.89, p = 0.309	**OR 0.9, p = 0.011**	**OR 0.9, p = 0.032**	**OR 0.9, p = 0.001**	OR 0.9, p = 0.122	OR 0.9, p = 0.113
	**CI0.8–0.9**	**CI 0.8–0.9**	CI 0.7–1.1	**CI 0.84–0.98**	**CI 0.9–1.0**	**CI 0.8–0.9**	CI 0.9–1.0	CI 0.9–1.0
**Baseline**	OR 1.36 p = 0.072	OR 1.34, p = 0.078	OR 1.17, p = 0.695	**OR 1.68, p<0.001**	**OR 1.84, p<0.001**	OR 1.19, p = 0.194	OR 1.1, p = 0.546	OR 1.1, p = 0.41
**EDSS**	CI 1.0–1.9	CI 1.0–1.9	CI 0.5–2.5	**CI 1.3–2.2**	**CI 1.4–2.5**	CI 0.9–1.5	CI 0.8–1.5	CI 0.8–1.6
**baseline**	OR 1.01 p = 0.334	OR 1.02, p = 0.096	OR 1.04, p = 0.222	**OR 1.02, p = 0.018**	**OR 1.02, p = 0.031**	OR 1.01, p = 0.48	OR 1.0, p = 0.733	OR 0.99, p = 0.558
**FSS**	CI 1.0–1.1 n = 221	CI 1.0–1.1 n = 228	CI 1.0–1.1 n = 226	**C I1.004–1.04 n = 221**	**CI 1.002–1.04 n = 215**	CI 1.0–1.1 n = 222	CI 0.9–1.0 n = 197	CI 0.9–1.0 n = 197
**Disease**	OR 1.03 p = 0.242	OR 1.03, p = 0.333	OR 1.03, p = 0.628	**OR 1.09, p = 0.001**	**OR 1.05, p = 0.04**	**OR 1.05, p = 0.031**	OR 1.01, p = 0.606	OR 1.04, p = 0.123
**duration**	CI 1.0–1.1 n = 220	CI 1.0–1.1 n = 228	CI 0.3–1.2 n = 226	**CI1.03–1.14 n = 220**	**CI 1.002–1.09 n = 215**	**CI 1.004–1.09 n = 222**	CI 1.0–1.1 n = 197	CI 1.0–1.1 n = 197
**progression**	**OR 3.68 p = 0.001**	**OR 2.70, p = 0.014**	OR 0.49, p = 0.4	**OR 9.3, p<0.001**	**OR 3.5, p = 0.002**	OR 1.7, p = 0.134	OR 1.2, p = 0.646	OR 1.5, p = 0.321
	**CI 1.7–8.2**	**CI 1.2–6.0**	CI 0.1–2.6	**CI 3.1–27.3**	**CI 1.6–8.0**	CI 0.9–3.4	CI 0.6–2.6	CI 0.7–3.3
**gender**	OR 0.62 p = 0.290	OR 0.66, p = 0.340	OR 0.50, p = 0.369	OR 1.05, p = 0.878	OR 1.1, p = 0.743	OR 0.75, p = 0.359	OR 1.01, p = 0.968	OR 1.07, p = 0.856
	CI 0.3–1.5	CI 0.3–1.5	CI 0.1–2.3	CI 0.6–1.9	CI 0.6–2.0	CI 0.4–1.4	CI 0.5–2.0	CI 0.5–2.2
**Δ EDSS**	**OR 1.42 p = 0.002**	**OR 1.35, p = 0.014**	OR 1.32, p = 0.357	**OR 1.70, p<0.001**	OR 1.15, p = 0.150	OR 1.21, p = 0.057	OR 1.23, p = 0.067	OR 1.03, p = 0.832
	**CI 1.11–1.82**	**CI 1.06–1.71**	CI 0.7–2.4 n = 226	**CI 1.4–2.1 n = 220**	CI 0.95–1.39	CI 0.9–1.5	CI 0.99–1.54	CI 0.8–1.3
**Δ FSS**	**OR 1.04 p = 0.003**	**OR 1.04, p = 0.002**	OR 1.04, p = 0.137	OR 1.01, p = 0.190	OR 1.00, p = 0.716	**OR 1.03, p = 0.003**	**OR 1.03, p = 0.015**	OR 1.01, p = 0.276
	**CI 1.01–1.06**	**CI 1.01–06.1 n = 225**	CI 0.99–1.1 n = 223	CI 0.99–1.03 n = 218	CI 0.98–1.01 n = 214	**CI 1.01–1.05 n = 219**	**CI 1.01–1.05 n = 194**	CI 0.99–1.03 n = 194

**Table 3:** Multivariate analysis verbal memory, sustained attention/information processing speed, executive function/conceptual reasoning.

VERBAL MEMORY			SUSTAINED ATTENTION/INFORMATION PROCESSING SPEED		EXECUTIVE FUNCTION/CONCEPTUAL REASONING		
	SRWL-IRn = 225	SRWL-DR n = 225	SDMT n = 218	PASAT n = 217	MCST-CATn = 218	MCST-PEn = 194	MCST-NPE n = 199
**Age**				adjOR 1.04, p = 0.004			adjOR 1.04, p = 0.048
				CI 1.01–1.08			CI 1.001–1.08
**AWM-f**	adjOR1.72, p<0.001	adjOR 1.61, p<0.001	adjOR1.49, p = 0.006	adjOR1.43, p = 0.002	adjOR 1.52,p<0.001	adjOR 1.51, p = 0.001	adjOR 1.26, p = 0.032
	CI 1.35–2.20	CI 1.28–2.03	CI 1.12–1.97	CI 1.14–1.80	CI 1.2–1.9	CI 1.2–1.9	CI 1.02–1.55
**Disease**			adjOR1.05, p = 0.069				
**duration**			CI 0.996–1.11				
**ΔFSS**	adjOR 1.04 p = 0.006	adjOR 1.04 p = 0.004			adjOR 1.03p = 0.004	adjOR 1.03, p = 0.016	
	CI 1.01–1.06	CI 1.01–1.06			CI 1.01–1.05	CI 1.005–1.05	
**Baseline EDSS**			adjOR1.66, p = 0.003				
			CI 1.19–2.32				
**Δ EDSS**			adjOR1.73, p<0.001				
			CI 1.4–2.2				

PASAT scores of 217 MS patients were evaluated. Twenty-four patients were excluded from the analysis because their PASAT scores were not available. Seventy-three percent of RR→SP and 43% of RR→RR patients’ scores were under cutoff (p = 0.002). PASAT showed a positive correlation with AWM-f (OR 1.43, CI 1.14–1.80 p = 0.002) and age (OR 1.04, CI 1.01–1.08 p = 0.004). In other words, for a 1% increase of AWM-f there was a 43% increased odds of having impaired PASAT scores. One year increase in age was related to 4% increased odds of impaired score at this test (see Table 3).

SRWL scores of 230 MS patients were evaluated. Eleven patients were excluded from the analysis because their SRWL scores were not available. Thirty-three per cent of RR→SP patients and 12% of RR→RR patients showed SRWL-immediate recall (IR) under cut off (p = 0.001). SRWL-IR score showed a positive correlation with AWM-f (OR 1.72 CI 1.35–2.20 p<0.001) and ΔFSS (OR 1.04 CI 1.01–1.06 p = 0.006) such that a 1% increase of AWM-f was related to 72% higher odds to be impaired at this test. ΔFSS was related to higher odds of impaired performances at this test. The SRWL-delayed recall (DR) was statistically different between 2 groups, with 30% RR→SP patients showing SRWL scores under cut off if compared to 14% RR→RR scores (p = 0.012). A multivariable analysis showed a positive correlation between SRWL-DR scores, AWM-f (OR 1.61 CI 1.28–2.03 p<0.001) and ΔFSS (OR 1.04 CI 1.01–1.06 p = 0.004).In other words, for a 1% increase of AWM-f there was a 61% increased odds to have SRWL-DR score under cut-off and ΔFSS was related to higher odds of impaired performances at this test (see Table 3).

MCST scores of 224 patients were evaluated. Seventeen patients were excluded from the analysis because their scores were not available. The MCST-category (CAT) scores of 49% of RR→SP and 34% of RR→RR patients were under cut off (p = 0.08). The multivariable analysis showed a positive correlation between MCST-CAT, AWM-f (OR 1.52, CI 1.2–1.9 p<0.001) and ΔFSS (OR 1.03 CI 1.01–1.05 p = 0.004). In other words, a higher AWM-f was related to a 52% increased odds of MCST-CAT impairment and ΔFSS was related to higher odds of impaired performances at this test. Out of 199 RR→RR patients only 30% and 24% showed perseverative (MCSTpe) and non perseverative errors (MCSTnpe) respectively. The multivariable logistic regression step-wise model showed a positive correlation between AWM-f, MCSTpe (OR 1.51 CI 1.2–1.9 p = 0.001) and ΔFSS (OR 1.03 CI 1.005–1.05 p = 0.016), such that a higher AWM-f was related to a 51% increased odds to be cognitively impaired at this test and ΔFSS was related to a higher odds of impaired performances at this test. Regarding MCSTnpe, a positive correlation was found with age (OR 1.04 CI 1.001–1.08 p = 0.048) and AWM-f (OR 1.26 CI 1.02–1.55 p = 0.032). This means that AWM-f was related to a 26% increased odds to be cognitively impaired at MCSTnpe and one year increase in age was related to a 4% increased odds of impaired performances at this test (see Table 3).

We evaluated cognitive performances of 227 patients. Out of them, 69 (28.6%) were cognitively impaired and 158 (69.6%) cognitively preserved. In particular the prevalence of CIm was 27.1% in RR→RR and 45% in RR→SP group (p = 0.025). As expected, cognitively impaired patients showed a significantly higher overall CIm than cognitively preserved patients (19.0±3.8 and 10.8±3.9, p<0.001). The multivariable logistic regression step-wise model regarding CIm showed a positive correlation with AWM-f (OR 1.63, CI 1.3–2.1, p>0.001) and disease duration (OR 1.04, CI 1.0–1.1, p = 0.06). A negative binomial regression analysis showed AWM-f was the only significant parameter showing an Incidence Rate Ratio of 1.09 (CI 1.06–1.12, p>0.001). In other words for a 1% increase of AWM-f there was a 9% increased overall CIm index score. Considering EDSS at baseline and FU visit we found median EDSS was 2.0 at BL and 3.0 at the end of FU (p < 0.001). Logistic regression showed a positive correlation between ΔEDSS and CIm (OR 1.29 CI 95% 1.05–1.57 p = 0.013): this means that one point increase in EDSS was related to 29% odds to be cognitively impaired. Considering FSS at baseline and FU, we found median FSS was 26 at BL and 37 at FU (p<0.001). Logistic regression showed a positive correlation between ΔFSS and CIm (OR 1.03 CI 95% 1.01–1.05 p = 0.004), such that one point increase of FSS was related to 3% odds to be cognitively impaired. Moreover, the multivariable analysis showed a positive correlation between ΔEDSS and SDMT (OR 1.73 p<0.001), and between ΔFSS, and SRWL-IR (OR 1.04 p = 0.006), SRWL-DR (OR 1.04 p = 0.004), MCSTCAT (OR 1.03 p = 0.004) and MCST-pe (OR 1.03 p = 0.01).

## Discussion

In this study we evaluated the role of WM, GM and LL (i.e. AWM-f) volumes in predicting the long-term occurrence of CIm in a large group of MS patients.

In the last years, it has been showed that CIm is more related to brain atrophy than to LL in mildly disabled MS patients [4, 8]. On the other hand, WM lesions seem to play an important role in the development of CIm as well [20, 21]. The contribution of WM damage to CIm is also confirmed by studies reporting a clear association between cognitive functioning and cortico–cortical and cortico–subcortical WM tracts damage [21, 22]. In particular, the association between the WM damage and the impairment of information processing speed, generally studied by PASAT and SDMT, is frequently demonstrated [23]. A 5 years-follow up study showed that brain atrophy is a good predictor of cognitive functioning in RRMS patients, although also T1-hypointense lesions showed a good predictive value [5]. This is in line with findings suggesting that atrophy of cortical and sub-cortical deep GM could be associated with WM lesion burden [24]. However, the pathophysiologic process remains poorly understood [25].

**Fig 2:**
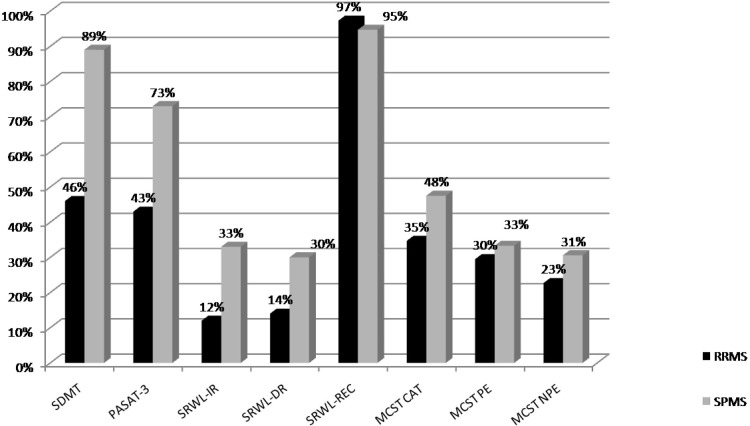
Proportion of RRMS and SPMS patients with impaired performance at each test. Legend: RRMS = relapsing-remitting multiple sclerosis, SPMS = secondary progressive multiple sclerosis, SDMT = symbol digit modality test, PASAT-3 = 3.0 inter-stimulus interval of Paced Auditory Serial Addition Test, SRWL = semantically related word list test, IR = immediate recall, DR = delayed recall, REC = recognition, MCST CAT = modified card sorting test categories, MCST-PE = MCST perseverative errors, MCST-NPE = MCST non-perseverative errors.

Using a fully automated segmentation method, we found that AWM-f, indicating LL, was the best predictor of CIm in MS patients. In particular, AWM-f was predictive of an impaired SDMT performance. The lower SDMT scores in RR→SP patients compared to RR→RR (proportion of RRMS and SPMS patients with impaired performance at each test represented in Fig. 2) and the positive correlation with EDSS at BL, underline the influence of accrual of disability and WM damage on cognitive performance. Since an interaction between motor disability and cognition has been demonstrated, especially on progressive patients, we cannot exclude that motor impairment may affect in part CIm [26]. However, our patients have a relatively mild disability, being baseline EDSS below 4 for both RRMS and SPMS (2.1 ± 0.9 and 3.17 ± 1 respectively). Our results showed higher PASAT scores in RR→RR if compared to RR→SP patients. In the multivariable analysis, age and AWM-f were the only variables predictive of lower PASAT scores, confirming previous findings showing that lower age was related to better performances at this test [27].Taken together, these results underline the importance of WM tract integrity in the rapid transfer of information between the cortex and deep GM, suggesting the involvement of WM in processing speed deficits.

Regarding MCST, we found a positive correlation with AWM-f. This is in line with other studies, showing that although MCST has a lower sensitivity than other executive function tests in MS, it is strongly correlated with brain atrophy and LL [28].

RR→SP showed lower SRWL scores when compared to RR→RR group. Moreover, we found a positive correlation between AWM-f and the incidence of being classified as impaired on the SRWL.

Our results could be apparently in contrast with other studies reporting that patients with progressive MS show deficits in information processing speed, attention, working memory, executive function, and verbal episodic memory, whereas CIm of RRMS patients is limited to information processing speed and working memory [29]. The association between verbal memory and GM pathology is frequently reported. Using a different battery (BRB) and a different MRI technique, Amato et al [30] showed a clear association between GM atrophy and verbal memory, suggesting the involvement of cortical regions in the neuropathological process at the early stage of the disease; however, given the cross sectional nature of the study, a direct comparison with the results presented herein is not possible. Memory deficits of MS patients were initially thought to be due only to impaired retrieval. More recent explanations postulate that verbal memory impairment is the consequence of an inadequate acquisition and retrieval, both secondary to information processing insufficiency [31], although it is conceivable the impairment of memory and information processing speed may result from the same pathological process. In line with this perspective, a robust correlation between CIm and LL is reported in a number of studies conducted with mildly disabled patients or at the early stage of the disease [32–34] Using high field MRI, a correlation between Normal Appearing WM, SDMT and CVLT-II [35] scores has been demonstrated, highlighting the crucial role of WM tract integrity in verbal learning, ensuring rapid transfer of information between cortex and deep GM. In our study, we support the role of a fully automated, operator-independent, multiparametric segmentation method to measure AWM-f, as marker of LL and both WM and GM volumes [10].MRI was performed 9 years before the NPS, which allows us to suppose that AWM-f could be considered an early predictor of CIm, strengthening the concept of a possible involvement of WM in the development of CIm. It is conceivable that CIm in our patients is, at least in part, caused by central neural pathways-disconnection. A “disconnection model” could interpret the involvement of multiple cognitive domains in this pathology as a series of disconnection syndromes affecting different cognitive networks. We propose that disruption of cortical WM tracts leads to reduced connectivity between cortico-cortical and cortico-subcortical cognitive processing regions, resulting in deficits in specific cognitive domains. On the other hand, this kind of model does not exclude GM pathology [3, 30] which may play a more important role as the disease progress [36]. Recently, a 13-year follow-up study showed that GM magnetization transfer ratio (MTR) was the only MRI predictor of global CIm, supporting the notion that GM plays a major role in the long-term development of CIm [37]. However, we cannot rule out that GM damage is secondary to WM damage, emphasizing the role of WM as an early marker of CIm.

Our study has the appeal of focusing on the long term predicting value of MRI parameters in a real life setting of MS management, being our cohort not in the early phase of the disease and having a relatively low disability.

However, our project has several limitations. First, though we had the great advantage to use the same (mobile) scanner in each participating center so that all patients shared the same protocol on the same scanner, we used a 1.0 Tesla MRI scanner which did not allow us to perform more advanced MRI measurements (i.e. WM tractography). Second, we did not have a baseline cognitive evaluation, therefore we could not assess the cognitive profile of patients at such time-point. Third we did not perform a FU MRI to assess the longitudinal change in brain volume. On the other hand, the use of an accurate and automatic segmentation method gave us the advantage to simultaneously assess LL, WM and GM volumes in a large population of patients and in a time-saving fashion. Finally, we did not use a universally accepted method to calculate the overall CIm, thus possibly underestimating the real burden of CIm. Since there is a lack of a standardized classification criteria, we used two different strategies to have an estimate of global CIm: a composite index score based on a graded system [19] and a domain specific CIm. Furthermore, since the number of test may influence cognitive outcome, we decided to use a brief NPS evaluation, investigating the most commonly affected cognitive domains in MS, giving us the opportunity to test a large number of patients [38].Although we used a brief NPS battery, not all patients completed tests. However, given the small number of missing data for every test (19 were excluded from SDMT analysis, 24 from PASAT analysis, 11 from SRWL analysis, 17 from MCST) we do not believe this may affect our results.

In conclusion, our findings suggest that WMLL, a reliable and relatively easy to acquire MRI parameter, may have a role in the pathology of CIm in MS patients and could be considered as an early predictor of future cognitive decline. Further longitudinal studies are needed to better clarify the relation between WMLL and GM damage. In particular, we suggest that the use of automated segmentation procedures might be useful for planning future studies focused on selecting the best parameters for monitoring cognitive decline in MS patients.
